# Unusual Evolution of Carotid Atherosclerosis in a Patient with Transient Ischemic Attack

**DOI:** 10.3390/life15060831

**Published:** 2025-05-22

**Authors:** Corina Cinezan, Camelia Bianca Rus, Ioana Tiberia Ilias, Alexandra Comanescu, Alexandra Cinezan

**Affiliations:** 1 Department of Medical Disciplines, Faculty of Medicine and Pharmacy, University of Oradea, 410073 Oradea, Romania; rus.cameliabianca@student.uoradea.ro (C.B.R.); ioana.ilias@didactic.uoradea.ro (I.T.I.); 2 Clinical County Emergency Hospital Bihor, 410169 Oradea, Romania; a.comanescu@uoradea.ro; 3 Doctoral School of Biological and Biomedical Sciences, University of Oradea, 410087 Oradea, Romania; 4 Department of Psycho-Neuroscience and Recovery, Faculty of Medicine and Pharmacy, University of Oradea, 410073 Oradea, Romania; 5 Faculty of Dental Medicine, Iuliu Hațieganu University of Medicine and Pharmacy of Cluj-Napoca, 400012 Cluj-Napoca, Romania; cinezan.alexandra@elearn.umfcluj.ro

**Keywords:** carotid stenosis, stroke, atherosclerosis, subclinical inflammation, dyslipidemia target, colchicine, periodontal disease

## Abstract

Here, we report an unusual case of a nonsmoker and hypertensive 72-year-old male who was admitted with a transient ischemic attack to the Neurology Department of Clinical County Emergency Hospital Bihor. He presented a first transient ischemic attack and paroxysmal atrial fibrilation 2 years before, when anticoagulation was started on top of his antihypertensive medication. At that time, carotid Doppler ultrasound revealed nonobstructive atherosclerosis and statin therapy was started, according to current guidelines, in order to lower the initial 70 mg/dL LDL-cholesterol level to under 55 mg/dL. Cardio-embolism was considered the mechanism of stroke at that time. Despite all the medication and the maintenance of LDL below 50 mg/dL, carotid atherosclerosis evolved to an important left internal carotid artery stenosis and transient ischemic attacks reappeared two years later. Carotid stenosis was then considered the most probable cause, although elucidating the exact mechanism was difficult. After medical treatment and subsequent endarterectomy, the patient had a good outcome. The progressive course of atheromatosis, despite maximal medication, urged us to look for further proper measures of prevention. No chronic disease was detected during the postoperative phase, except for early-stage periodontal disease, for which adequate preventive measures were applied. Considering that subclinical inflammation induced by periodontal disease can induce the progression of atherosclerosis, chronic treatment with colchicine was added, with a favorable outcome.

## 1. Introduction

Stroke is a disabling disease that reduces quality of life [1,2]. Transient ischemic attack is a type of stroke with transitory functional disability but with the same prognostic importance. It can precede or follow a stroke and thus is an important alarm signal. Regarding secondary prevention, the approach to a patient with transient ischemic attack is similar to that for a patient with stroke [1,2,3,4,5,6].

When faced with a stroke patient, it is important to elucidate its mechanism in order to treat it according to guidelines and to prevent a new one [1,5]. The most common mechanism of stroke is cardio-embolism, mainly due to atrial fibrillation. A cause of at least equal importance is carotid atherosclerosis [5,6,7]. Atherosclerosis is the basis of many cardiovascular diseases [1,2]. In order to reduce cardiovascular mortality and morbidity, it is necessary to control its risk factors. It can also be the hidden hallmark of a systemic disease, and a multidisciplinary approach is necessary to reduce its consequences [3,4].

The treatment of a transient ischemic attack or stroke patient can be a real challenge in clinical practice [1,2]. Despite a considered effective treatment according to the guidelines, patients return with a second episode quite often [1,7,8]. Making a decision regarding treatment, and especially secondary prevention, can be difficult [1].

## 2. Detailed Case Description

We present a case of a 72-year-old nonsmoker and hypertensive man who was admitted with two episodes of global aphasia to The Neurology Department of the Clinical County Emergency Hospital Bihor. Each episode lasted about 10 min, followed by complete remission. The clinical examination revealed normal weight, teguments, osteoarticular, muscular, digestive, and respiratory systems, with normal findings. The blood pressure was 180/103 mmHg and the heart rate was 46 bpm. No cardiac and vascular murmurs were detected. The neurological exam was also normal. The laboratory exam is shown in Table 1.

**Figure 1:**
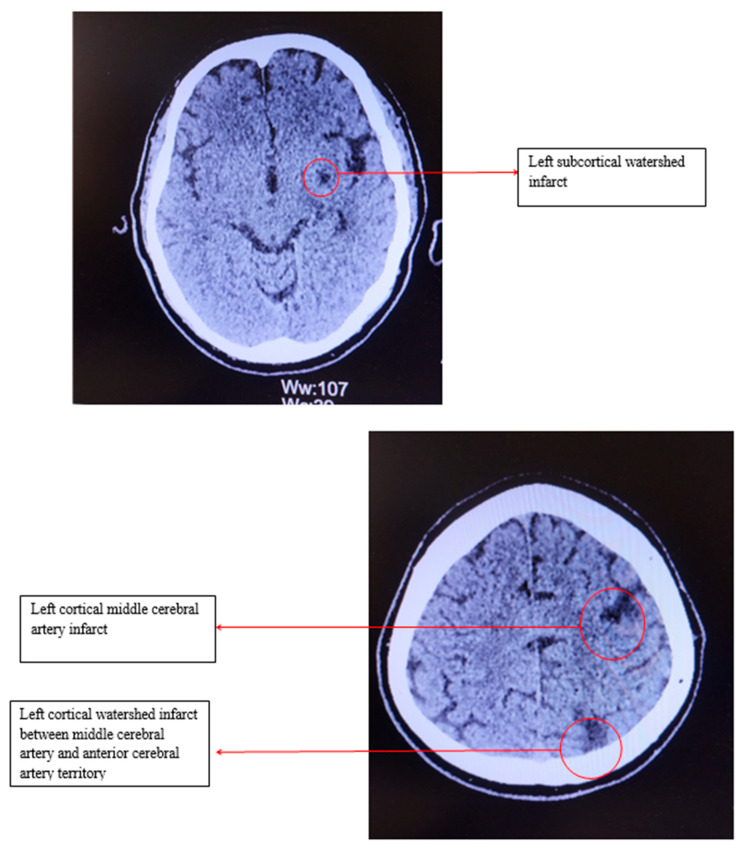
Computed tomography of the head.

Electrocardiography revealed sinus bradycardia, with a heart rate of 46 bpm and no ST/T changes. Cranial computed tomography showed several chronic infarction areas in the left frontal, left parietal, and in the left internal capsule (Figure 1).

The medical history of this patient is interesting. Two years ago, the patient presented at the emergency department after a short episode of aphasia, with complete remission. Physical examination was normal, and the computed tomography showed an old stroke and small vessel disease changes due to hypertension. The case was interpreted as a transient ischemic attack; the patient was not admitted to hospital and was deferred to ambulatory evaluation. It is noteworthy that he occasionally—three times a week—played tennis, with a good effort tolerance. His medication for hypertension was Olmesartan 40 mg/day, Amlodipine 10 mg/day, and Rilmenidine 1 mg/day, which he had been taking for four years, and his blood pressure was maintained between 120 and 130 systolic and 75–80 diastolic. The laboratory results from that time are shown in Table 2.

**Figure 2:**
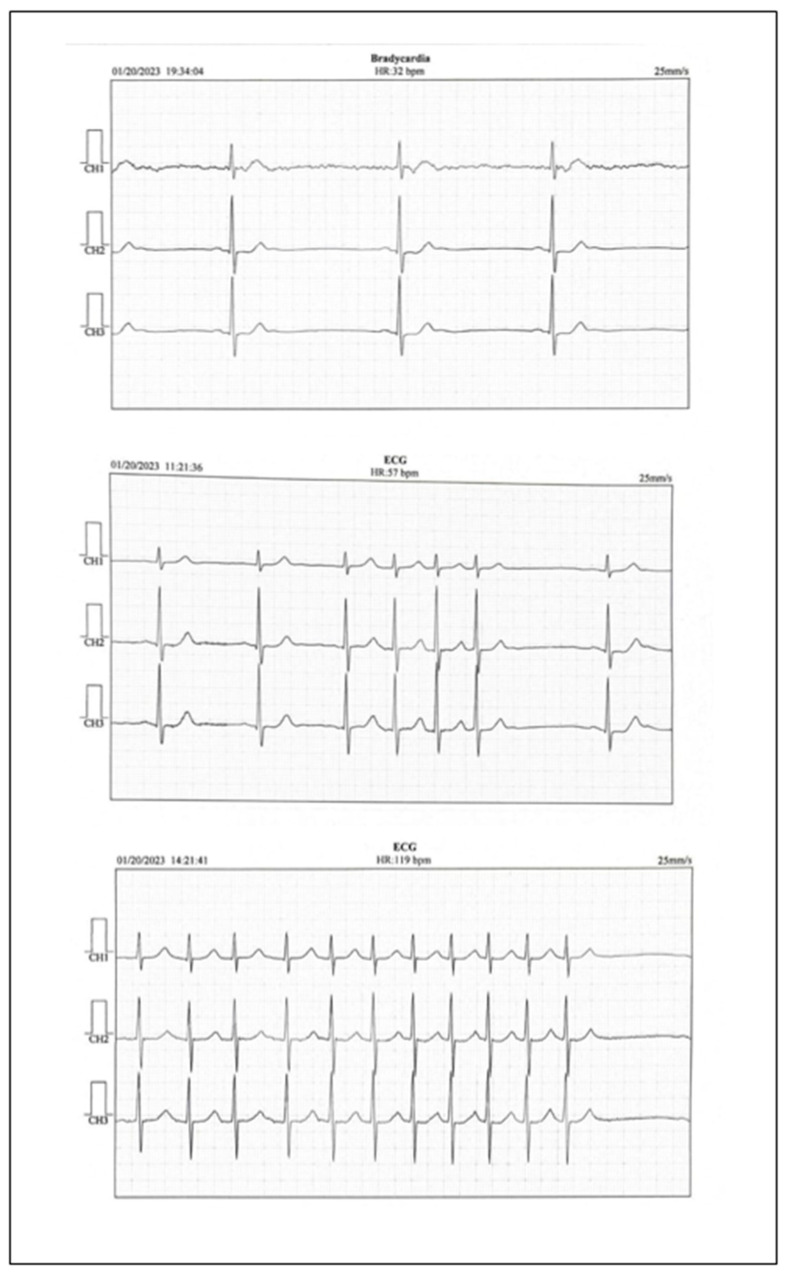
Holter EKG report.

The electrocardiogram revealed sinus bradycardia with a rate of 50 bpm. The echocardiography showed a slightly increased left atrium volume, a slight left ventricular hypertrophy with a normal left ventricular ejection fraction. No thrombus was detected in the left atrium. Abdominal ultrasound did not reveal pathological changes. The Doppler carotid ultrasound showed slight nonobstructive atherosclerosis of the left and right internal carotid arteries and no changes in the peripheral arteries. Analyzing the clinical symptoms, together with the paraclinical findings, left atrium volume, the normal LDL-cholesterol levels, the slight atherosclerosis of both internal carotid arteries, and the findings of the cerebral computed tomography, ambulatory electrocardiographic monitoring revealed sinus bradycardia with a minimum heart rate of 32 bpm, even in the daytime, and short episodes of atrial fibrillation with a high ventricular rate (Figure 2).

It is worth noting that after a slight daytime effort, the ventricular rate grew gradually to 100–110 bpm. The patient received 5 mg apixaban twice a day (CHA_2_DS_2_-VA score 4) and 20 mg atorvastatin once a day on top of his chronic treatment, to prevent future episodes of embolic ischemic attacks and to lower the LDL cholesterol to under 55 mg/dL, as needed according to guidelines. The patient received follow-up appointments in the neurological and cardiological outpatient department three times/year over the following two years. Lower heart rates, usually below 40 bpm were observed, although the patient was symptom free. The patient was proposed a pacemaker, but he refused it, because he had a good effort tolerance and his heart rate grew during practicing tennis. The LDL cholesterol levels were lowered to under 55 mg/dL and the treatment was maintained during the following two years.

**Figure 3:**
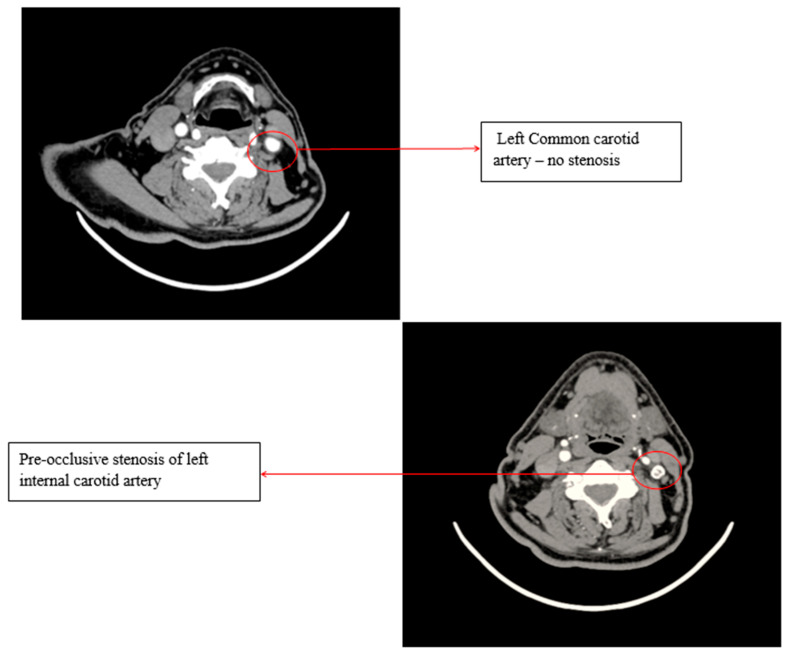
Computed tomography angiography (CTA) of the supra-aortic arteries.

A Doppler ultrasound of the precerebral vessels, performed during the present admission, revealed a pre-occlusive stenosis of the left internal carotid artery, which was not present two years ago. This stenosis was confirmed by computed tomography angiography of the supra-aortic arteries (Figure 3).

Transient ischemic attacks appeared, despite correct anticoagulation and LDL-cholesterol levels under 50 mg/dL. The left internal carotid stenosis was most probably the cause. Aspirin was added to the treatment and the dose of atorvastatin was increased to a maximum of 80 mg, given the pleiotropic effects of statin in atherosclerosis.

Keeping in mind the severity of carotid stenosis, the patient was deferred to vascular surgery.

Electrocardiogram was monitored for 24 h before the surgery, showing a minimum heart rate of 32 bpm in the night and 42 bpm during the daytime. Echocardiography looked almost the same as the old one and transesophageal echocardiography did not show any thrombus in the left atrium, nor in the left atrial appendage. The patient agreed to pacemaker insertion, but the multidisciplinary team, consisting of the neurologist, cardiologist, anesthesiologist, and vascular surgeon decided that the procedure ought to be postponed because of the risk of stroke. However, it is known that the manipulation of the carotid artery during the surgery could produce further bradycardia, but it is expected to be responsive to atropine, as it is responsive to effort. The patient was prepared for surgery and apixaban was replaced by enoxaparin for 48 h.

**Figure 4:**
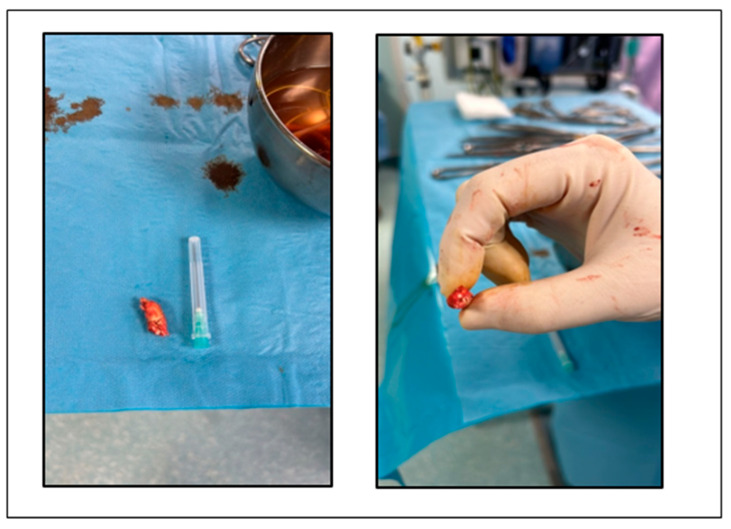
Atheroma removed from left internal carotid artery.

The patient underwent endarterectomy of the left internal carotid artery (Figure 4) under general anesthesia. During the intervention, a third degree sinoatrial block was observed, with heart rate of 26 bpm. After intravenous atropine, the normal sinus rhythm reappeared.

The postoperative evolution was very good and after a week, a pacemaker was implanted. On top of his treatment, including apixaban, the patient received clopidogrel 75 mg/zi. He will be reevaluated by vascular surgeon, cardiologist, and neurologist once every 3 months for the first year and then annually.

After a month of recovery, the patient was referred to the internist for evaluation of a possible chronic disease which could be a trigger for the evolution of his atherosclerotic carotid disease. Laboratory tests, which included tests for inflammatory markers and lipoprotein (a), chest radiography, abdominal ultrasound, gastroscopy, and colonoscopy did not reveal the presence of such a condition. The same happened in the case of the otorhinolaryngological examination.

The dental evaluation revealed the presence of gingivitis, the first stage of periodontal disease, in which only the soft tissue of the gum is affected and the patient may be asymptomatic. The patient followed the preventive treatment according to the dentist’s instructions. Considering the presence of periodontal disease as a cause of subclinical inflammation which could be involved in the mechanism of progression of atherosclerosis, we decided to add colchicine treatment. The ultrasound evaluation one year after the surgery did not reveal the evolution of carotid atherosclerosis, so the patient will follow chronic anti-inflammatory treatment, on top of his anti-hypertensive, anticoagulant, antiplatelet, and high-dose statin therapy.

## 3. Discussion

Treating a patient with a history of atrial fibrillation and ischemic stroke, despite oral anticoagulation, is challenging. First of all, we had to discover the potential mechanism of the second stroke in order to prevent the third [5,6,7,8].

Our patient presented to the emergency department after a second transient ischemic attack. One in every five strokes is caused by atrial fibrillation. This patient’s medical history included atrial fibrillation and, according to the guidelines, the risk of ischemic stroke was substantially elevated, and the subject had to be evaluated regarding the CHA_2_DS_2_-VA score [5]. This score includes congestive heart failure (one point), hypertension (one point), age ≥ 75 years (two points), diabetes mellitus (one point), prior stroke/transient ischemic attack/arterial thromboembolism (two points), vascular disease (one point), and age 65–74 years (one point) [5,6]. Our patient had a score of 4 and received apixaban, together with his antihypertensive medication and a statin, because nonobstructive carotid atherosclerosis was present, intending to lower the cardiovascular risk together with the LDL cholesterol to under 55 mg/dL. Despite receiving anticoagulant and statin, he had a second transient ischemic attack. It is worth noting that no significant vascular disease was diagnosed at that time.

The possible mechanism of stroke in this type of patient includes a competing mechanism, other than atrial fibrillation-related embolism; non-adherence to anticoagulants or an insufficient dose; or cardio-embolism related or not to atrial fibrillation [1,7,8]. Among 2946 patients, Polymeris et al. [7] reported a competing mechanism in 24%, insufficient anticoagulation in 32%, and cardio-embolism despite anticoagulation in 44%. In our patient, a competing mechanism of transient ischemic attack is a more reasonable explanation, keeping in mind the positive result of computed tomography angiography and the negative one of transesophageal echocardiography.

Another challenge in this case is the evolution of carotid atherosclerosis, despite the levels of LDL cholesterol being lower than 50 mg/dL after statin treatment [2,5]. Amarenco et al. [9] concluded that after an ischemic stroke—including transient ischemic attack—patients treated with statins who had an LDL cholesterol target of less than 70 mg/dL had a lower risk of subsequent cardiovascular events than those treated with an LDL cholesterol target of 90 to 110 mg/dL. In our patient, carotid atherosclerosis developed with an LDL cholesterol level below the target. Considering the unilateral presence of the chronic neurological condition, we agree that the atherosclerotic mechanism could have been involved from the beginning in the etiology of the stroke and that perhaps we should have initiated antiplatelet therapy at that time, in addition to anticoagulant and statin therapy. Maybe other factors were implicated in this evolution [3,4,10,11].

It is important to say that the same experienced neurologist performed both carotid echographies. He used the NASCET (North American Symptomatic Carotid Endarterectomy Trial) criteria based on peak systolic velocity in the carotid arteries to diagnose severe carotid artery stenosis [10]. According to the guidelines [10,11], the patient has to receive dual antiplatelet therapy with aspirin and clopidogrel, and carotid endarterectomy is indicated within 14 days because medical therapy alone is not sufficient to prevent stroke in patients with significant carotid stenosis [10,11,12,13,14].

During carotid endarterectomy, transitory bradycardia without hemodynamic compromise appeared, due to intraoperative carotid manipulation [13]. Lauro et al. [13] reported that intraoperative bradycardia and hypotension not responding to atropine and requiring vasopressors during and after carotid surgery were associated with major cardiovascular events. Our patient responded to intraoperative atropine and was hemodynamically stable during the postoperative period. Cardiovascular monitoring in the first 24 h after surgery is needed [13]. After surgery, the patient received a pacemaker, as indicated.

According to guidelines [10,11], aspirin or clopidogrel is recommended for a long time after internal carotid artery revascularization. It is necessary to assess neurological and cardiological symptoms, risk factors for atherosclerosis, hematological, renal and hepatic parameters, and adherence to therapy [9,10,11,12]. The hypertension and dyslipidemia should be treated properly, to lower the risk of future cardiovascular events [9,10,11,12,14]. Our patient needs oral anticoagulation, antiplatelet therapy, and a more careful assessment, because of their higher risk of bleeding.

The most important issue in this case was the unknown mechanism of the evolution of carotid atherosclerosis. From this point of view, we considered subclinical inflammation as a mechanism of atherosclerosis evolution, meaning that maximum dose of statin in the treatment of this patient was desirable, due to its pleiotropic effect, even though the LDL cholesterol value was under 50 mg/dL with a lower dose [3,4,7,9]. Knowing that inflammation of the arterial wall may play a role in the evolution of atherosclerosis, anti-inflammatory therapy would be pivotal [3,4,15,16]. Maybe a lower LDL cholesterol target is needed for certain individuals [14]. High plasma concentration of oxidized LDL is associated with all stages of atherosclerosis and with the magnitude of its complications [16]. Given the logistic issues, we could not determine this parameter in our patient. We must take into account other risk factors for atherosclerosis than the classic ones too. Lipoprotein (a) should be one of them [17]. In this case, lipoprotein (a) was in the normal range.

As the patient did not have any apparent inflammatory disease, he was recommended an evaluation for an autoimmune or other disease, even though he did not have any symptoms, with negative results [3,4]. A dental evaluation was desirable too, knowing that periodontitis is one of the most common underdiagnosed inflammatory disease and is associated with an increased cardiovascular risk [18]. After the detection of the first stage periodontal disease, the patient was treated by a dentist in order to prevent the evolution of his dental problem. In this case, the only explanation for the development of atherosclerosis was subclinical inflammation in the context of periodontal disease, and we choose to add colchicine to the chronic treatment because, in current medicine, colchicine has been proven to have an important role in the management of atherosclerosis [10,19,20].

Development of atherosclerotic plaque is an inflammatory process [1,2]. Arterial inflammation was found to be associated with traditional risk factors for atherosclerosis, like hypertension, male sex, advanced age, smoking, and obesity, three of these factors being present in our patient [1,2,3,4]. Active arterial inflammation was found to precede the development of atherosclerotic plaque and is an early stage of atherosclerosis [1,2]. Oxidative stress, endothelial disfunction, lipid metabolism, and immune responses are associated mechanisms in the development and progression of atherosclerosis [15,16,21,22]. A total of 60% of participants in the Progression of Early Subclinical Atherosclerosis (PESA) Study [16] classified as low cardiovascular risk according to traditional atherosclerosis risk factors showed evidence of subclinical atherosclerosis. Other authors [22] imagined a ‘’systemic inflammatory response index’’, based on a computed, composed value of neutrophils, monocytes, and lymphocytes. This index was elevated in people who developed carotid atherosclerosis, even though they did not have a history of hypertension, diabetes, or dyslipidemia.

These are the reasons why we considered subclinical inflammation caused by periodontal disease as a treatment target in our patient.

## 4. Conclusions

There is always room for an unexpected evolution in the medical field, even though the current therapy runs according to guidelines. Atherosclerosis underlies many cardiovascular events and is one of the most surprising entities in medicine. Not all of its manifestations are known yet. To treat and prevent future cardiovascular events, in this patient and perhaps in all, a multidisciplinary approach is mandatory, taking into account all clinical features, mechanisms, and classical and novel risk factors for atherosclerosis and atherothrombosis.

## Figures and Tables

**Table 1:** Blood and urine tests—neurology admission.

Test	Result	Reference Range
White Blood Cell Count	9.81 10^3^/uI	4.00–10.0 10^3^/uI
Hemoglobin	14.1 g/dL	12.6–17.4 g/dL
Platelet Count	203 g/dL	150–450 g/dL
Blood glucose	94 mg/dL	82–115 mg/dL
Total Cholesterol	117 mg/dL	0–200 mg/dL
Triglycerides	108 mg/dL	0–150 mg/dL
High-Density Lipoprotein Cholesterol	49 mg/dL	40–60 mg/dL
Low-Density Lipoprotein Cholesterol	48.71 mg/dL	10–100 mg/dL
Aspartate Transaminase	30 U/L	5–34 U/L
Alanine Transaminase	13 U/L	0–55 U/L
Total Bilirubin	0.51 mg/dL	0.2–1.2 mg/dL
Serum Urea	25.00 mg/dL	8.4–2.57 mg/dL
Serum Creatinine	1.47 mg/dL	0.72–1.25 mg/dL
Serum Uric Acid	7.00 mg/dL	3.5–7.2 mg/dL
C-Reactive Protein	4.60 mg/L	0–5.0 mg/L
Potassium	4.50 mmol/L	3.50–4.50 mmol/L
Sodium	140 mmol/L	135–145 mmol/L
Glomerular Filtration Rate	51 mL/min/1.73 m^2^	90–120 mL/min/1.73 m^2^
Microalbuminuria	10 mg/L	0–10 mg/L
Urinary Sediment Test	Negative	Negative

**Table 2:** Blood and urine tests—cardiology admission.

Test	Result	Reference Range
White Blood Cell Count	8.56 10^3^/uI	4.00–10.0 10^3^/uI
Hemoglobin	15.6 g/dL	12.6–17.4 g/dL
Platelet Count	197 g/dL	150–450 g/dL
Blood Glucose	96 mg/dL	82–115 mg/dL
Total Cholesterol	146 mg/dL	0–200 mg/dL
Triglycerides	75 mg/dL	0–150 mg/dL
High-Density Lipoprotein Cholesterol	61 mg/dL	40–60 mg/dL
Low-Density Lipoprotein Cholesterol	70 mg/dL	10–100 mg/dL
Aspartate Transaminase	25 U/L	5–34 U/L
Alanine Transaminase	15 U/L	0–55 U/L
Total Bilirubin	0.70 mg/dL	0.2–1.2 mg/dL
Serum Urea	22.00 mg/dL	8.4–2.57 mg/dL
Serum Creatinine	1.41 mg/dL	0.72–1.25 mg/dL
Serum Uric Acid	7.00 mg/dL	3.5–7.2 mg/dL
C-Reactive Protein	2.20 mg/L	0–5.0 mg/L
Glomerular Filtration Rate	54 mL/min/1.73 m^2^	90–120 mL/min/1.73 m^2^
Microalbuminuria	9 mg/L	0–10 mg/L
Urinary Sediment Test	Negative	Negative

## Data Availability

The raw data supporting the conclusions of this article will be made available by the authors on request. The data are not publicity available due to privacy reasons.
